# Supplementary material for: Body Mass Index during Gluten-Free Diet in Patients with Celiac Disease
Source: Nutrients. 2023 Aug 10;15(16):3517. doi: 10.3390/nu15163517 (PMC10457784; doi:10.3390/nu15163517)
Supplement: Supplementary file 1 [file nutrients-15-03517-s001.zip › nutrients-2496720-supplementary.pdf]

Supplementary Table S1. STROBE checklist.

	Item No.	Recommendation	Page No.	Relevant text from manuscript
**Title and abstract**	1	(*a*) Indicate the study's design with a commonly used term in the title or the abstract	1	
		(*b*) Provide in the abstract an informative and balanced summary of what was done and what was found	1	
**Introduction**				
Background/rationale	2	Explain the scientific background and rationale for the investigation being reported	1-2	
Objectives	3	State specific objectives, including any prespecified hypotheses	2	
**Methods**				
Study design	4	Present key elements of study design early in the paper	2	
Setting	5	Describe the setting, locations, and relevant dates, including periods of recruitment, exposure, follow-up, and data collection	2-4	
Participants	6	(*a*) *Cohort study*—Give the eligibility criteria, and the sources and methods of selection of participants. Describe methods of follow-up<br>*Case-control study*—Give the eligibility criteria, and the sources and methods of case ascertainment and control selection. Give the rationale for the choice of cases and controls<br>*Cross-sectional study*—Give the eligibility criteria, and the sources and methods of selection of participants	2-3	
		(*b*) *Cohort study*—For matched studies, give matching criteria and number of exposed and unexposed<br>*Case-control study*—For matched studies, give matching criteria and the number of controls per case		
Variables	7	Clearly define all outcomes, exposures, predictors, potential confounders, and effect modifiers. Give diagnostic criteria, if applicable	3-4	
Data sources/ measurement	8*	For each variable of interest, give sources of data and details of methods of assessment (measurement). Describe comparability of assessment methods if there is more than one group	3-4	
Bias	9	Describe any efforts to address potential sources of bias		
Study size	10	Explain how the study size was arrived at	2-3	

Continued on next page

Quantitative variables	11	Explain how quantitative variables were handled in the analyses. If applicable, describe which groupings were chosen and why	4
Statistical methods	12	(*a*) Describe all statistical methods, including those used to control for confounding	4
		(*b*) Describe any methods used to examine subgroups and interactions	4
		(*c*) Explain how missing data were addressed	
		(*d*) *Cohort study*—If applicable, explain how loss to follow-up was addressed<br>*Case-control study*—If applicable, explain how matching of cases and controls was addressed<br>*Cross-sectional study*—If applicable, describe analytical methods taking account of sampling strategy	
		(*e*) Describe any sensitivity analyses	
**Results**			
Participants	13*	(a) Report numbers of individuals at each stage of study—eg numbers potentially eligible, examined for eligibility, confirmed eligible, included in the study, completing follow-up, and analysed	3
		(b) Give reasons for non-participation at each stage	3
		(c) Consider use of a flow diagram	3
Descriptive data	14*	(a) Give characteristics of study participants (eg demographic, clinical, social) and information on exposures and potential confounders	4-6
		(b) Indicate number of participants with missing data for each variable of interest	
		(c) *Cohort study*—Summarise follow-up time (eg, average and total amount)	4
Outcome data	15*	*Cohort study*—Report numbers of outcome events or summary measures over time	4-10
		Case-control study—Report numbers in each exposure category, or summary measures of exposure	
		Cross-sectional study—Report numbers of outcome events or summary measures	
Main results	16	(*a*) Give unadjusted estimates and, if applicable, confounder-adjusted estimates and their precision (eg, 95% confidence interval). Make clear which confounders were adjusted for and why they were included	
		(*b*) Report category boundaries when continuous variables were categorized	
		(*c*) If relevant, consider translating estimates of relative risk into absolute risk for a meaningful time period	

Continued on next page

Other analyses	17	Report other analyses done—eg analyses of subgroups and interactions, and sensitivity analyses	4-10
**Discussion**			
Key results	18	Summarise key results with reference to study objectives	10-12
Limitations	19	Discuss limitations of the study, taking into account sources of potential bias or imprecision. Discuss both direction and magnitude of any potential bias	12
Interpretation	20	Give a cautious overall interpretation of results considering objectives, limitations, multiplicity of analyses, results from similar studies, and other relevant evidence	12
Generalisability	21	Discuss the generalisability (external validity) of the study results	12
**Other information**			
Funding	22	Give the source of funding and the role of the funders for the present study and, if applicable, for the original study on which the present article is based	12

*Give information separately for cases and controls in case-control studies and, if applicable, for exposed and unexposed groups in cohort and cross-sectional studies.

Note: An Explanation and Elaboration article discusses each checklist item and gives methodological background and published examples of transparent reporting. The STROBE checklist is best used in conjunction with this article (freely available on the Web sites of PLoS Medicine at http://www.plosmedicine.org/, Annals of Internal Medicine at http://www.annals.org/, and Epidemiology at http://www.epidem.com/). Information on the STROBE Initiative is available at www.strobe-statement.org.

Supplementary Table S2. Characteristics of the 17 CD patients included in the analysis depicted in Figure 3.

Age at diagnosis of CD	Gender	Clinical presentation of CD	Mean BMI at diagnosis of CD (kg/m^2)	BMI class at diagnosis of CD	Mean BMI at short-term follow-up (kg/m^2)	BMI class at short-term follow-up	Mean BMI at intermediate-term follow-up (kg/m^2)	BMI class at intermediate-term follow-up	Mean BMI at long-term follow-up (kg/m^2)	BMI class at long-term follow-up
29	Female	Classical	19.5	Normal	28.5	Overweight	27.3	Overweight	28.9	Overweight
26	Female	Classical	16.0	Underweight	18.8	Normal	18.8	Normal	18.4	Underweight
38	Female	Non-classical	20.4	Normal	20.8	Normal	20.8	Normal	20.8	Normal
35	Female	Classical	16.4	Underweight	20.5	Normal	21.1	Normal	18.9	Normal
51	Female	Classical	17.8	Underweight	23.2	Normal	22.7	Normal	21.2	Normal
47	Male	Non-classical	21.7	Normal	24.0	Normal	27.5	Overweight	26.3	Overweight
64	Female	Non-classical	23.3	Normal	22.7	Normal	21.5	Normal	22.9	Normal
49	Male	Classical	19.5	Normal	19.5	Normal	20.6	Normal	20.9	Normal
45	Female	Non-classical	20.6	Normal	20.6	Normal	20.3	Normal	21.3	Normal
43	Female	Non-classical	20.1	Normal	20.1	Normal	21.4	Normal	20.8	Normal
56	Female	Classical	24.9	Normal	25.3	Overweight	27.1	Overweight	28.4	Overweight
35	Female	Non-classical	19.8	Normal	20.8	Normal	20.8	Normal	22.3	Normal
40	Female	Classical	20.5	Normal	22.0	Normal	25.1	Overweight	25.9	Overweight
48	Female	Non-classical	19.7	Normal	19.7	Normal	20.3	Normal	19.7	Normal
62	Female	Classical	18.4	Underweight	19.9	Normal	20.7	Normal	20.3	Normal
34	Female	Non-classical	27.1	Overweight	29.0	Overweight	28.6	Overweight	30.3	Obesity class I.
67	Female	Classical	27.2	Overweight	29.6	Overweight	28.4	Overweight	28.0	Overweight

CD: celiac disease; BMI: body mass index.
